# Yersinia antarctica sp. nov., isolated from an Antarctic insect – expanding the genus into polar ecosystems

**DOI:** 10.1099/ijsem.0.007268

**Published:** 2026-08-10

**Authors:** Anne-Sophie Le Guern, Matan Shelomi, Tetiana Bahlai, Laura Guichard, Carine Martins, Javier Pizarro-Cerda, Cyril Savin

**Affiliations:** 1 Institut Pasteur, Université de Paris Cité, Yersinia Research Unit, National Reference Center for Plague & Other Yersinioses, WHO Collaborating Centre for Plague FRA-146, F-75015 Paris, France; 2 Department of Entomology, National Taiwan University, No. 1, Sec. 4, Roosevelt Rd., Taipei 106319, Taiwan, ROC; 3 National Antarctic Scientific Center of Ukraine, 16 blvd. Tarasa Shevchenka, Kyiv 01601, Ukraine

**Keywords:** insect, MALDI-TOF, phylogeny, *Yersinia*

## Abstract

Five Gram-negative strains, designated BI01^T^, BI02, BI1211, BI4D and BI67 isolated from the gut of the Antarctic endemic insect *Belgica antarctica*, were characterized by a polyphasic approach. Comparative 16S rRNA gene sequences indicated that the bacterial strains belonged to the genus *Yersinia*. A cgMLST-based phylogeny based on 500 concatenated core genes strongly supported a clade encompassing the 5 strains that was distinct from the currently recognized 27 species of the genus *Yersinia*. Average nucleotide identity (ANI) among the five strains consistently exceeded 99%, but comparisons to their closest relative, *Yersinia intermedia*, yielded an ANI of 93% and a digital DNA–DNA hybridization value of 51.74. Metabolic profiling further differentiated the five strains from other closely related species within the genus *Yersinia*, notably with respect to l-fucose and potassium 2-ketogluconate fermentation, acetoin production and an absence of pyrazinamidase activity. Interestingly, three of the five strains exhibited resistance to *β*-lactams (amoxicillin and amoxicillin/clavulanate) and first- and second-generation cephalosporins (cefalexin and cefoxitin) associated with the presence of the *ampC* gene, encoding a class C beta-lactamase, in their genome. None of the five isolates harbour the classic virulence determinants of enteropathogenic *Yersinia*. The DNA G+C content of the five strains is 47.8 mol%. Based on these results, they represent a novel species of the genus *Yersinia*, for which the name *Yersinia antarctica* sp. nov. is proposed. Notably, this represents the first *Yersinia* species reported from Antarctic terrestrial invertebrates. The type strain is BI01^T^ (=CIP 112622^T^=DSM 121309^T^). The distribution of this species is not limited to Antarctica, as few publicly available genomes indicate its presence in continental Russia and the Russian Arctic, with isolates recovered from rodents, polar bears and birds.

## Introduction

The genus *Yersinia* comprises Gram-negative, facultatively anaerobic bacteria within the family *Yersiniaceae* and encompasses 27 species of significant ecological and clinical importance [1–3], according to the List of Prokaryotic names with Standing in Nomenclature accessed in January 2026 [4]. While *Yersinia pestis* is responsible for plague, a fatal vector-borne disease transmitted from rodent reservoir to humans by fleas, *Yersinia pseudotuberculosis* and *Yersinia enterocolitica* are human enteropathogens found in diverse animal reservoirs; notably, *Y. enterocolitica* colonizes pig tonsils without causing disease [5, 6]. Interestingly, *Yersinia entomophaga* and *Yersinia ruckeri*, which are basal species within the *Yersinia* genus, are pathogens of insects and fishes, respectively [7, 8]. The genus also includes numerous environmental taxa isolated from water and soil [6, 9]. A main phenotypic characteristic of *Yersinia* is psychrotolerance, allowing survival and proliferation at low temperatures, facilitating persistence in cold environments [10, 11]. This trait poses great challenges for the food industry and public health, as it can lead to transfusion-transmitted bacteraemia [12, 13].

Polar ecosystems harbour a wide diversity of microbial taxa shaped by low temperatures, freeze–thaw cycles, oligotrophy and episodic nutrient inputs [14, 15]. In Antarctica, terrestrial arthropods show low species diversity and are highly specialized, and their microbiomes remain underexplored relative to those of temperate insects [16, 17]. To our knowledge, there are no published studies on *Yersinia* ecology in polar areas, although 16S rRNA gene sequences of *Yersinia* have been identified from the *Colobanthus quitensis* plant in Antarctica and from arctic stream sediments in Alaska [18, 19]. This gap in the literature leaves open questions about the diversity, adaptation and evolutionary relationships of *Yersinia* lineages in these extreme environments.

In a recent investigation of the gut microbes associated with the endemic Antarctic midge, *Belgica antarctica*, we isolated a group of five bacterial strains that exhibited distinctive physiological and genomic features (Table 1). The microbes were originally isolated from the digestive tracts of field-collected *B. antarctica* larvae dissected under sterile conditions at the Vernadsky Research Base on Galindez Island off the Antarctic Peninsula. Phylogenomic analyses demonstrated that these strains form a coherent and distinct clade within the *Yersinia* genus. Based on these findings, we suggest the classification of these strains as a novel species, for which the name *Yersinia antarctica* sp. nov. is proposed [20]. This is likely the correct species name for putative *Yersinia* identified in prior whole-genome sequencing data from *B. antarctica* [21]. Here, we present a comprehensive description of the phenotypic and genomic characteristics of *Y. antarctica* sp. nov., including its differentiation from recognized *Yersinia* species.

**Table 1.** Origin of the strains belonging to the novel *Yersinia* species

Strain	Collection no.	Proposed novel species	Isolation						
			Source	Origin	Continent	Area	Latitude	Longitude	Isolation date
BI01^T^	CIP 112622^T^ DSM 121309^T^	*Y. antarctica*	*B. antarctica*	Gut	Antarctica	Galindez Island	−65.2500	−64.2500	18-01-2025
BI02	CIP 112623 DSM 121310	*Y. antarctica*	*B. antarctica*	Gut	Antarctica	Galindez Island	−65.2500	−64.2500	18-01-2025
BI1211	CIP 112624 DSM 121311	*Y. antarctica*	*B. antarctica*	Gut	Antarctica	Berthelot Island	−65.3333	−64.1500	26-01-2025
BI4D	CIP 112643 DSM 121312	*Y. antarctica*	*B. antarctica*	Gut	Antarctica	Cape Tuxen	−65.2667	−64.1333	04-02-2025
BI67	CIP 112650 DSM 121313	*Y. antarctica*	*B. antarctica*	Gut	Antarctica	Yalour Island	−65.2345	−64.1625	07-02-2025

## Phenotypic characterization

Cells were cultured aerobically for 48 h at 28 °C on tryptic soy agar (TSA) (Bio-Rad), cefsulodin–irgasan–novobiocin (CIN) agar (Oxoid) and CHROMagar^™^ *Y. enterocolitica* (CHROMagar). Gram staining was performed from TSA-grown colonies, and cellular morphology was examined by light microscopy. Psychrotolerance was assessed by streaking isolates, pre-cultured in Luria-Bertani (LB) broth for 24 h at 28 °C, onto TSA agar plates. The plates were incubated at 4 °C, and colony sizes were measured daily for 10 days.

Biochemical characteristics were determined at 28 °C using API 20E strips (bioMérieux) for *β*-galactosidase, arginine dihydrolase, lysine decarboxylase, ornithine decarboxylase, citrate utilization, H_2_S production, urease, tryptophan deaminase, indole production, acetoin production (Voges–Proskauer) and gelatinase, and using API 50CH strips (bioMérieux) for carbohydrate fermentation (under anaerobic conditions). Catalase activity was determined by bubble formation in 3% (v/v) hydrogen peroxide. Motility and nitrate reductase were tested on mannitol-motility-nitrate (MMN) semisolid medium (Clearline) incubated at 28 °C for 24 h. To detect nitrate reductase activity, three drops of reagent A (sulfanilic acid) and three drops of reagent B (α-naphthylamine) were added to the surface of the medium. Oxidase activity was tested using tetramethyl-p-phenylenediamine–impregnated discs (Sigma-Aldrich). Tween esterase activity was detected on agar containing Tween 80, with a positive reaction indicated by aggregate formation in the medium [22]. Pyrazinamidase activity was evaluated on pyrazinamide-containing agar, with a positive reaction indicated by a brown colouration in the presence of ferrous salts [23]. Strains were serotyped using a panel of four O-antigen–specific rabbit antisera [O:3 (Bio-Rad), O:5, O:8 and O:9 (in-house)] associated with virulence. The characteristics of the proposed novel species are detailed in the species description.

Distinctive phenotypic traits (l-fucose and potassium 2-ketogluconate fermentation, acetoin production and pyrazinamidase activity) differentiating the putative novel species from all other described *Yersinia* species and subspecies (excluding *Y. pestis*) were identified by comparison with type strains and additional reference isolates phenotypically characterized at the French Yersinia National Reference Center (Tables 2a and 3b and Fig. S1, available in the online Supplementary Material) [2].

**Table 2.** (a) Distinctive biochemical characteristics between the novel *Y. antarctica* species and the other *Yersinia* species (*n*=26) and subspecies (except *Y. pestis*)

**Species**	*Y. antarctica*	*Y. aldovae*	*Y. aleksiciae*	*Y. alsatica*	*Y. artesiana*	*Y. bercovieri*	*Y. canariae*	*Y. enterocolitica*						*Y. entomophaga*	*Y. fenwickii*	*Y. frederiksenii*
								BT 1A*	BT 1B	BT2	BT3	BT4	BT5			
**Number of strains**	5	2	6	9	4	10	2	27	4	58	23	30	4	2	9	4
API 20E results																
*β*-Galactosidase	+	d	+	+	+	+	+	+	+	+	+	+	d	+	+	+
Lysine decarboxylase	−	−	−	−	−	−	−	−	−	−	−	−	−	−	−	−
Ornithine decarboxylase	d	+	+	d	d	−	−	d	d	d	d	−	−	+	−	d
Citrate utilization	−	d	−	d	−	−	−	−	−	−	−	−	−	+	d	−
Urease	−	+	+	+	+	+	+	+	+	+	+	+	+	−	+	+
Indole	+	−	−	+	+	−	+	+	−	+	−	−	−	−	+	+
Acetoin production	+	+	−	+	+	−	−	+	+	+	d	+	d	+	d	+
API 50CH results																
l-Arabinose	d	+	+	+	+	+	+	+	+	+	+	+	+	−	+	+
d-Ribose	+	+	+	+	+	+	+	+	+	+	+	+	+	+	+	+
d-Xylose	+	+	+	+	+	+	+	+	+	+	+	−	+	−	+	+
d-Adonitol	−	−	−	−	−	−	−	−	−	−	−	−	−	−	−	−
l-Sorbose	+	−	−	+	+	−	+	+	+	+	+	+	d	−	+	+
l-Rhamnose	−	+	−	+	−	−	−	−	−	−	−	−	−	−	−	+
inositol	+	+	+	+	+	−	+	+	−	+	d	−	−	−	+	+
d-Sorbitol	+	+	+	+	+	+	+	+	+	+	+	+	d	−	+	+
Arbutin	+	−	+	+	+	d	+	+	+	d	d	d	d	−	+	+
Aesculin ferric citrate	d	−	d	+	+	−	+	+	−	−	−	−	−	−	+	+
Salicin	+	−	−	+	+	d	+	d	−	d	−	−	d	−	+	+
d-Cellobiose	+	−	d	+	+	+	+	+	+	+	+	+	+	d	+	+
d-Maltose	+	−	+	+	+	+	+	+	+	+	+	+	d	+	+	+
d-Lactose	d	−	d	−	+	d	+	+	d	+	d	−	d	d	d	−
d-Melibiose	d	−	−	−	−	−	−	−	−	−	−	−	−	+	+	−
d-Sucrose	+	−	−	+	+	+	+	+	+	+	+	+	+	+	+	+
d-Trehalose	+	+	+	+	+	+	+	+	+	+	+	+	d	+	+	+
d-Raffinose	−	−	−	−	−	−	−	−	−	−	−	−	−	+	+	−
Starch	−	−	−	−	−	−	−	−	−	−	−	−	−	−	−	−
Gentiobiose	+	−	+	+	+	+	+	+	+	+	+	+	d	−	+	+
d-Tagatose	−	−	−	−	−	−	−	−	−	−	−	−	−	−	−	−
l-Fucose	+	+	d	+	−	+	+	+	−	−	−	−	−	−	+	+
d-Arabitol	−	−	+	+	+	−	−	d	−	−	−	−	−	−	+	+
Potassium gluconate	+	−	+	+	+	+	+	+	+	d	+	−	−	+	+	+
Potassium 2-ketogluconate	−	−	−	−	+	−	−	−	−	−	−	−	−	−	d	−
Potassium 5-ketogluconate	+	−	+	+	+	+	+	+	+	d	d	−	−	+	+	+
Lipase activity	+	+	−	+	−	−	+	+	+	−	−	−	−	+	d	+
Motility at 28 °C	+	−	d	+	−	d	−	+	+	+	d	−	−	+	+	+
Pyrazinamidase activity	−	+	+	+	+	+	−	+	−	−	−	−	−	+	d	+

In red, unique combination of biochemical characteristics allowing to identify *Y. antarctica* species.

*, BT means biotype.

+, 90%+, 90% or more strains positive.

−, 90%−, 90% or more strains negative.

d, 11–89% of strains positive.

**Table 3.** (b) Distinctive biochemical characteristics between the novel *Y. antarctica* species and the other *Yersinia* species (*n*=26) and subspecies (except *Y. pestis*)

Species	*Y. hibernica*	*Y. intermedia*	*Y. kristensenii*	*Y. massiliensis*	*Y. mollaretii*	*Y. nurmii*	*Y. occitanica*	*Y. pekkanenii*	*Y. proxima*	*Y.* *pseudotuberculosis*	*Y. rohdei*	*Y. ruckeri*	*Y. similis*	*Y. thracica*	*Y. vastinensis*	*Y. wautersii*
Number of strains	2	6	4	6	11	1	6	1	10	11	6	3	4	4	5	4
API 20E results																
*β-Galactosidase*	+	+	+	+	+	+	+	+	+	+	+	+	d	d	+	+
Lysine decarboxylase	–	–	–	–	–	–	–	–	–	–	–	+	–	–	–	–
Ornithine decarboxylase	d	+	d	+	d	+	+	–	d	–	–	+	–	d	d	–
Citrate utilization	–	d	–	–	–	+	d	–	d	–	–	–	–	–	–	–
Urease	+	+	+	+	+	–	+	+	+	+	+	–	+	+	+	+
Indole	–	+	+	+	–	–	d	–	+	–	d	–	–	–	+	–
Acetoin production	–	+	–	d	–	+	–	–	+	–	–	–	–	–	+	–
API 50CH results																
l-Arabinose	+	+	+	+	+	–	+	+	+	+	+	–	+	+	+	+
d-Ribose	+	+	+	+	+	+	+	+	+	d	+	+	d	+	+	d
d-Xylose	+	+	+	+	+	–	+	+	+	+	+	–	+	+	+	+
d-Adonitol	–	–	–	–	–	–	–	–	–	d	–	–	–	–	–	d
l-Sorbose	+	+	+	+	+	–	d	–	+	–	–	–	–	+	+	–
l-Rhamnose	–	+	–	–	–	–	–	–	–	+	–	–	+	–	+	+
inositol	+	+	d	d	d	–	d	–	+	–	d	–	–	d	+	–
d-Sorbitol	+	+	+	+	+	–	+	–	+	–	+	d	–	+	+	–
arbutin	+	+	+	+	+	–	d	–	+	+	+	–	+	d	+	+
Aesculin ferric citrate	+	+	–	+	+	–	–	–	+	+	–	–	+	–	+	+
Salicin	+	+	–	+	+	–	–	–	+	d	+	–	d	–	+	d
Cellobiose	+	+	+	+	+	–	+	+	+	–	+	–	–	+	+	–
Maltose	+	+	+	+	+	+	+	+	+	+	+	+	+	+	+	+
Lactose	–	–	d	–	–	–	d	–	+	–	–	–	–	d	+	–
Melibiose	–	+	–	–	–	–	–	–	–	+	+	–	–	–	–	+
Sucrose	–	+	–	+	+	+	–	–	+	–	+	–	–	–	+	–
Trehalose	+	+	+	+	+	+	+	+	+	+	+	+	+	+	+	+
Raffinose	–	+	–	–	–	–	–	–	–	–	+	–	–	–	–	+
Starch	–	–	–	–	–	–	–	–	–	–	–	–	d	–	–	–
Gentiobiose	+	+	+	+	+	–	+	+	+	–	+	–	–	+	+	–
d-Tagatose	–	–	–	–	–	–	–	–	–	–	–	–	–	–	–	d
l-Fucose	–	+	+	+	–	–	–	–	–	–	–	–	–	–	+	–
d-arabitol	+	–	+	+	d	–	–	+	+	+	–	–	d	–	+	+
Potassium gluconate	+	+	+	+	+	–	+	–	+	+	+	–	d	d	+	d
Potassium 2-ketogluconate	–	–	–	–	–	–	d	–	–	–	d	–	–	–	–	–
Potassium 5-ketogluconate	+	+	+	+	+	–	+	–	+	d	+	–	–	–	+	d
Lipase activity	+	+	+	–	–	+	–	–	+	–	–	+	–	d	d	–
Motility at 28 °C	–	d	d	d	+	+	d	–	+	d	d	+	d	d	d	–
Pyrazinamidase activity	+	+	+	+	+	+	+	+	+	–	+	+	d	+	+	–

In red, unique combination of biochemical characteristics allowing to identify *Y. antarctica* species.

+, 90%+, 90% or more strains positive.

−, 90%−, 90% or more strains negative.

d, 11–89% of strains positive.

The five strains were also characterized using MALDI-TOF MS. Sample preparation was performed using the Ethanol/Formic Acid Extraction Sample Preparation Procedure. Mass spectra were generated using a MALDI Biotyper Sirius (Bruker Daltonics, Germany). Data were analysed in automatic mode using Bruker FlexControl 3.4 software and MBT HT RUO 5.1 with an integrated taxonomy library (database BDAL V13.0.0.0 containing 12,438 reference spectra). Depending on the strain, the best match of the protein patterns was either *Y. bercovieri* (scores of 2.1, 2.04 and 2.08 for strains BI01^T^, BI02 and BI1211, respectively), * Y. kristensenii* (score of 2.04 for strain BI4D) or *Y. intermedia* (score of 2.16 for strain BI67). Finally, using MALDI-TOF MS, these strains cannot be distinguished from their genetically closest relative, *Y. intermedia*.

The antibiotic resistance profiles of the five strains were determined by the disc diffusion method (Bio-Rad) on Mueller-Hinton II agar according to the European Committee on Antimicrobial Susceptibility Testing (https://www.eucast.org) recommendation with an incubation temperature of 28 °C, as previously described [24]. E-TEST (bioMérieux) have been used according to the manufacturer’s instructions to determine the MIC of antimicrobial with resistance. Testing of clinically relevant antibiotics revealed that the BI02, BI1211 and BI4D strains were resistant to specific *β*-lactams: amoxicillin (MIC >256 mg l^−1^), amoxicillin/clavulanic acid (MIC >256 mg l^−1^) and first- and second-generation cephalosporins cefalotin (MIC >256 mg l^−1^) and cefoxitin (MIC >256 mg l^−1^). In contrast, strains BI01^T^ and BI67 were susceptible to amoxicillin (MIC=0.25 mg l^−1^), amoxicillin/clavulanic acid (MIC=0.25 mg l^−1^), cefalotin (MIC=4 mg l^−1^) and cefoxitin (MIC=3 mg l^−1^). All five strains were susceptible to ticarcilline, cefotaxime, ceftazidime, ciprofloxacin, nalidixic acid, cotrimoxazole (trimethoprim–sulfamethoxazole), tetracycline, ertapenem and amikacin.

## Genomic characterization

Growth of the five strains was performed in 5 ml LB broth at 28 °C under agitation (150 r.p.m.) for 24 h and was followed by genomic DNA extraction on Maxwell CSC 48 (Promega, Madison, USA) using the Maxwell RSC Cultured Cells DNA kit (reference AS1620, Promega). The genome sequence of the type strain BI01^T^ was determined by Plasmidsaurus using Oxford Nanopore Technology (PromethION instrument). Following sequencing, the bottom 5% of lowest-quality FASTQ reads were removed using Filtlong v0.2.1 with default parameters. The remaining reads were downsampled to 250 Mb to produce a rough sketch assembly with Miniasm v0.3. From the Miniasm assembly, the reads were re-downsampled to approximately 100X coverage, excluding low-quality reads (filtlong --mean_q_weight 10). The high-quality reads were then assembled with Flye v2.9.1 [25] using parameters for high-quality ONT reads, and the assembly was polished with Medaka v1.8.0 with high-quality reads. The BI01^T^ genome was assembled into 4 contigs, with the largest contig measuring 4,966,176 bp, and an average coverage of 96X. The total size reaches 5,086,312 bp, and DNA G+C content is 47.8 mol%.

The genome sequences of the four other strains (BI02, BI1211, BI4D and BI67) were generated as previously described [26], with libraries prepared using the Nextera XT DNA Library Preparation Kit (Illumina) and sequencing performed on an Illumina NextSeq 550, and a *de novo* assembly using SPAdes version 3.15.0 [27]. On average, genomes of the 4 studied strains were assembled into 136 contigs (min: 84; max: 206) with a total size of 4.96 Mb (min: 4.84; max: 5.12), an N50 value of 57,880 (min: 43,932; max: 86,877) and a DNA G+C content of 47.74 mol% (min: 47.7; max: 4.82).

Genome assembly statistics for the five strains were calculated using CheckM2 v1.0.2, indicating 100% completeness for all assemblies and contamination ranging from 0.00% to 0.09% (Table 4).

**Table 4.** Genomes’ characteristics of the five strains of the novel *Yersinia* species

Strain	BI01^T^	BI02	BI1211	BI4D	BI67
Accession number	JBSJQF000000000	JBSJQG000000000	JBSJQH000000000	JBSJQI000000000	JBSJQJ000000000
Genome length (bp)	5,086,312	5,119,914	4,884,802	4,842,179	4,982,416
Genome coverage	96X	84X	206X	143X	110X
Sequencing technology	Oxford Nanopore Technology	Illumina	Illumina	Illumina	Illumina
Assembled contigs	4	281	149	214	274
Contig N50 (bp)	4,966,176	43,932	86,877	52,554	48,155
CheckM2 completeness	100.0	100.0	100.0	100.0	100.0
CheckM2 contamination	0.09	0.09	0.0	0.01	0.08
DNA G+C content (mol%)	47.8	47.7	47.7	47.7	47.8
Total genes	4,714	4,859	4,564	4,522	4,705
Protein-coding genes	4,598	4,760	4,462	4,423	4,606
rRNA (5S, 16S, 23S)	22	14	13	13	13
tRNA	81	77	77	74	74
ncRNA	13	10	12	12	12

The 16S rRNA gene sequences of the five strains (BI01^ᴛ^, BI02, BI1211, BI4D and BI67) were amplified with primers 27F and 1492R as described by Lane [28] and deposited in GenBank. Accession numbers for BI01^T^, BI02, BI1211, BI4D and BI67 are PX427238, PX427239, PX427240, PX427259 and PX427266, respectively. Sequence similarity analysis of the 16S rRNA gene sequences using blast indicated that the five strains clustered within the genus *Yersinia*, with the highest similarity of 98.91–99.48% to * Y. kristensenii*.

**Fig. 1.**
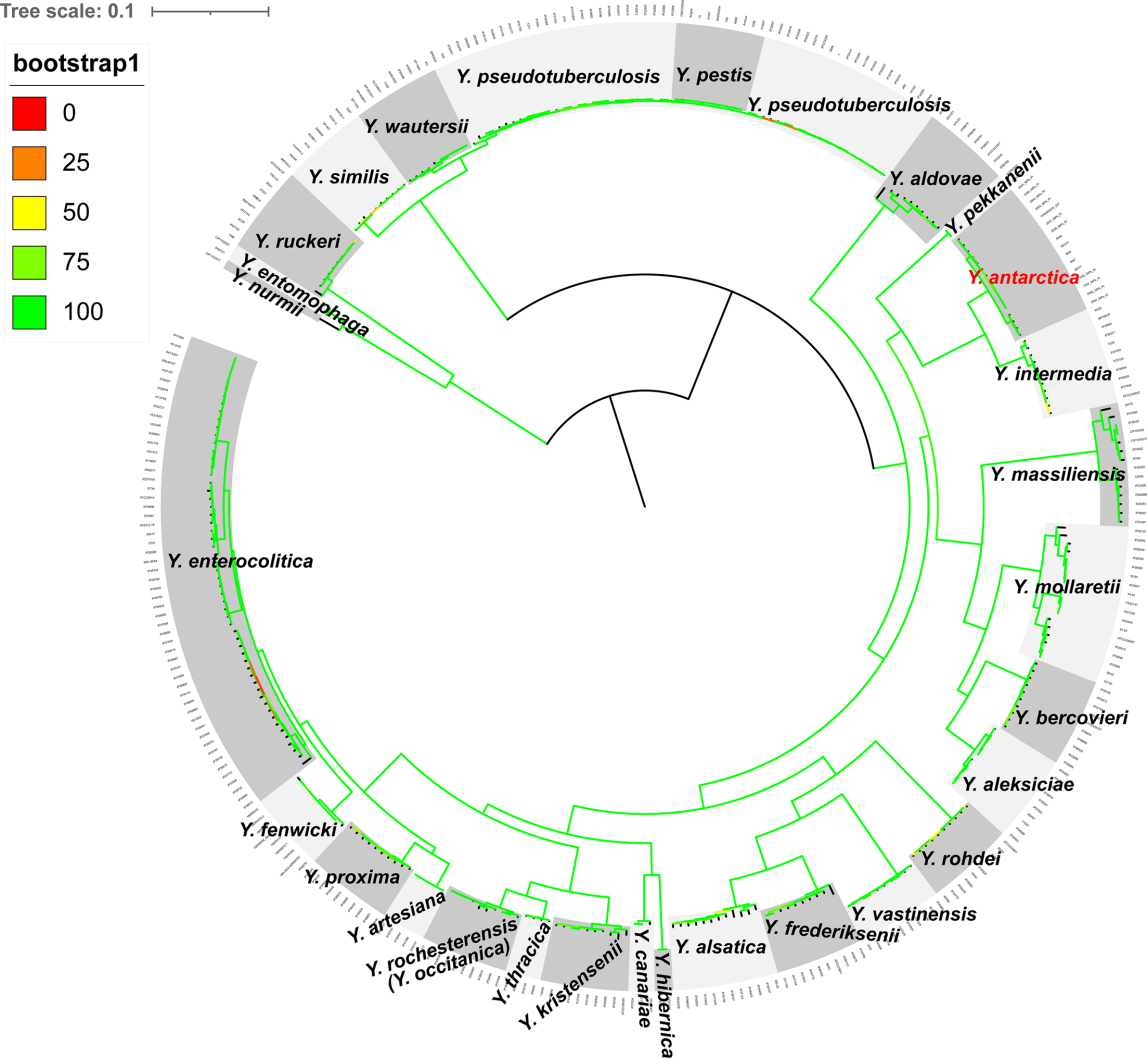
Maximum-likelihood phylogenetic tree of the genus *Yersinia* (322 strains) based on 500 concatenated gene multiple sequence alignments. Tree visualization was created with iTOL v7 [31]. Bootstrap support values are depicted using a colour gradient. The clade corresponding to ‘*Y. antarctica*’ is highlighted in red.

A phylogenetic analysis was performed using the concatenated nucleotide sequences of the 500 genes of the cgMLST *Yersinia* [26] with 333 strains belonging to the 27 species of *Yersinia* [2], including the very recently described * Y. fenwicki* [3]. We included 11 publicly available genomes putatively assigned to this novel *Yersinia* species. These genomes originate from strains isolated from rodents, polar bears and birds across continental Russia and the Russian Arctic region (Table S1). Concatenated sequences were aligned using MAFFT v7.467, and a maximum-likelihood phylogenetic reconstruction was performed using IQ-TREE v2.4.0 with ModelFinder to estimate the best variant of the General Time Reversible model [29, 30]. Tree visualization was created with iTOL v7 [31] (Figs 1 and S2). The five strains fall into a well-demarcated new clade close to *Y. intermedia*.

Pairwise average nucleotide identity (ANI) values among all strains included in the phylogenetic reconstruction were computed with fastANI v1.34 [32]. The 5 strains, together with the 11 publicly available genomes putatively assigned to this novel *Yersinia* species, shared a mean ANI of 98.83% (range: 97.67–99.95%), whereas ANI to other *Yersinia* species ranged from 80.80% to 93.75% (Table S2), with *Y. intermedia* being the closest relative (Fig. S3). These results confirm that the 16 strains constitute a novel *Yersinia* species, given the proposed ANI species-delineation threshold of 95–96% [32]. Digital DNA–DNA hybridization (dDDH) values between the 16 strains and the 11 genomes of their closest relative, *Y. intermedia*, were calculated using the Genome-to-Genome Distance calculator hosted by the Leibniz Institute DSMZ (https://ggdc.dsmz.de/) [33]. We report results based on Formula 2, as it is robust when using draft genomes. The mean dDDH values are 51.63 (50.5–52.8%) (Table S3), which is well below the 70% threshold for species delineation [34], thus confirming that the 16 strains represent a novel *Yersinia* species.

Screening the five genomes for antimicrobial resistance determinants using AMRFinderPlus version 4.2.7 (option: -i 0.8) [35] detected the presence of the *ampC* gene, encoding a class C beta-lactamase, in the three genomes exhibiting phenotypic resistance to *β*-lactams and cephalosporins, suggesting that these resistances are mediated by the expression of this gene [36]. The *ampC* gene was also detected in the 11 publicly available genomes putatively assigned to this novel *Yersinia* species. The expression of *ampC* is inducible, and its temperature-dependent modulation results in higher levels of resistance at an environmental temperature of 22 °C compared with 30 °C or 37 °C [37]. Analysis of the *ampC* genomic context in the three beta-lactam-resistant isolates revealed a conserved arrangement wherein *ampC* is flanked upstream by the transcriptional regulator *ampR* and downstream by a gene encoding an MBL-fold metallohydrolase. In contrast, the susceptible strain BI01^T^ lacks this three-gene cluster, and strain BI67 retains only *ampR*. These patterns suggest specific deletion events in the susceptible strains, occurring within a broader genomic context that remains conserved across all five strains.

A total of 42 virulence-associated genes were identified in the genomes of the 5 isolates using the Virulence Factor Database [38] implemented in ABRicate v 1.0.1 (https://github.com/tseemann/abricate). Among these, the chemotaxis operon components *cheA*, *cheB*, *cheD*, *cheR*, *cheW*, *cheY* and *cheZ* were identified, indicating the bacteria’s capacity to sense and adapt to fluctuations in chemical environmental composition. Flagellar virulence determinants were also identified, including the sigma factor *fliA* and the structural gene clusters *fliE-fliJ*, *fliL-fliN*, *fliP-fliT*, *fliZ*, *flgA-flgD*, *flgF-flgK*, *flgM*, *flgN* and *flhA-flhD*. Additionally, the *motA* gene encoding the flagellar motor protein MotA, *yapC* encoding a putative autotransporter protein homologous to the enterotoxigenic *Escherichia coli* adhesin TibA and *tssD* encoding a component of a type VI secretion system injection apparatus were identified [39–41]. None of the five isolates harbour the classic virulence determinants of enteropathogenic *Yersinia*, such as the *ail* and *inv* genes or the type III secretion system encoded by the *Yersinia* virulence plasmid [6], nor did they contain the *YenTc* toxin gene cluster implicated in the insect pathogenicity of *Y. entomophaga* [42].

## Description of *Yersinia antarctica* sp. nov.

*Yersinia antarctica* (ant.arc'ti.ca. L. fem. adj. *antarctica*, southern, Antarctic, where the type strain BI01^T^ was isolated).

Cells are short, Gram-negative rods. On CIN agar, colonies are circular with a large, deep-red centre surrounded by a translucent, pale border; they measure 1.0–1.5 mm and 3.0–3.5 mm in diameter after 24 and 48 h of incubation, respectively. On TSA, colonies are circular, beige, smooth and convex, measuring 0.5–1.0 mm at 24 h and 2.0–3.0 mm at 48 h. On CHROMagar, colonies are circular, 0.5–1.0 mm at 24 h and 1.5–2.0 mm at 48 h; they appear beige to light blue at 24 h, turning dark blue by 48 h. The psychrotolerance of the five strains was confirmed by the formation of pinpoint colonies after 7 days of incubation at 4 °C, which increased to 1 mm in diameter after 10 days. The five strains are motile on MMN semisolid medium and reduce nitrate. Oxidase is negative, and catalase is positive. Tween esterase is positive, and pyrazinamidase is negative. In API20E tests performed at 28 °C, all strains are positive for *β*-galactosidase, tryptophan deaminase (weak), indole and acetoin production (Voges–Proskauer), and negative for arginine dihydrolase, lysine decarboxylase, citrate utilization, H_2_S production, urease and gelatinase for all strains. Four out of the five isolates, including the type strain, are negative for ornithine decarboxylase. In API 50CH tests performed at 28 °C, acid production from glycerol, ribose, d-xylose, galactose, d-glucose, d-fructose, d-mannose, l-sorbose, inositol, mannitol, sorbitol, *N*-acetylglucosamine, arbutin, salicin, cellobiose, maltose, sucrose, trehalose, gentiobiose, l-fucose and potassium 5-ketogluconate is positive, and no acid production from erythritol, d-arabinose, l-xylose, adonitol, methyl *β*-d-xylopyranoside, rhamnose, dulcitol, methyl *α*-d-mannoside, methyl *α*-d-glucoside, amygdalin, inulin, melezitose, d-raffinose, starch, glycogen, xylitol, d-lyxose, d-tagatose, d-fucose, d-arabitol, l-arabitol or gluconate and potassium 2-ketogluconate. All strains are not typeable for the O:antigen with the four antisera associated with virulence.

The type strain, BI01^T^ (=CIP 112622^T^=DSM 121309^T^), as well as strains BI02, BI1211, BI4D and BI67, was isolated from the gut of the insect *B. antarctica*. The distribution of this species is neither restricted to the insect *B. antarctica* nor limited to the Antarctic region; indeed, publicly available genomes confirm its presence across continental Russia and the Russian Arctic, with isolates recovered from diverse hosts, including rodents, polar bears and birds. The complete genome of BI01^T^ together with the four other strains (BI02, BI1211, BI4D and BI67) has been deposited in the National Center for Biotechnology (https://www.ncbi.nlm.nih.gov/) database under the BioProject ID PRJNA1344266 and accession numbers SRX31151122–SRX31151126. The DNA G+C content of the type strain is 47.8 mol%.

## Supplementary material

10.1099/ijsem.0.007268 Supplementary Material 1.

10.1099/ijsem.0.007268 Supplementary Material 2.

## Abbreviations

ANI: average nucleotide identity
CIN: cefsulodin–irgasan–novobiocin
dDDH: digital DNA–DNA hybridization
LB: Luria-Bertani
MMN: mannitol-motility-nitrate
TSA: tryptic soy agar
