# Supplementary material for: Yersinia antarctica sp. nov., isolated from an Antarctic insect – expanding the genus into polar ecosystems
Source: Int J Syst Evol Microbiol. 2026 Aug 10;76(8):007268. doi: 10.1099/ijsem.0.007268 (PMC13456053; doi:10.1099/ijsem.0.007268)
Supplement: Supplementary file 1 [file ijsem-76-07268-s001.pdf]

Figure S1:

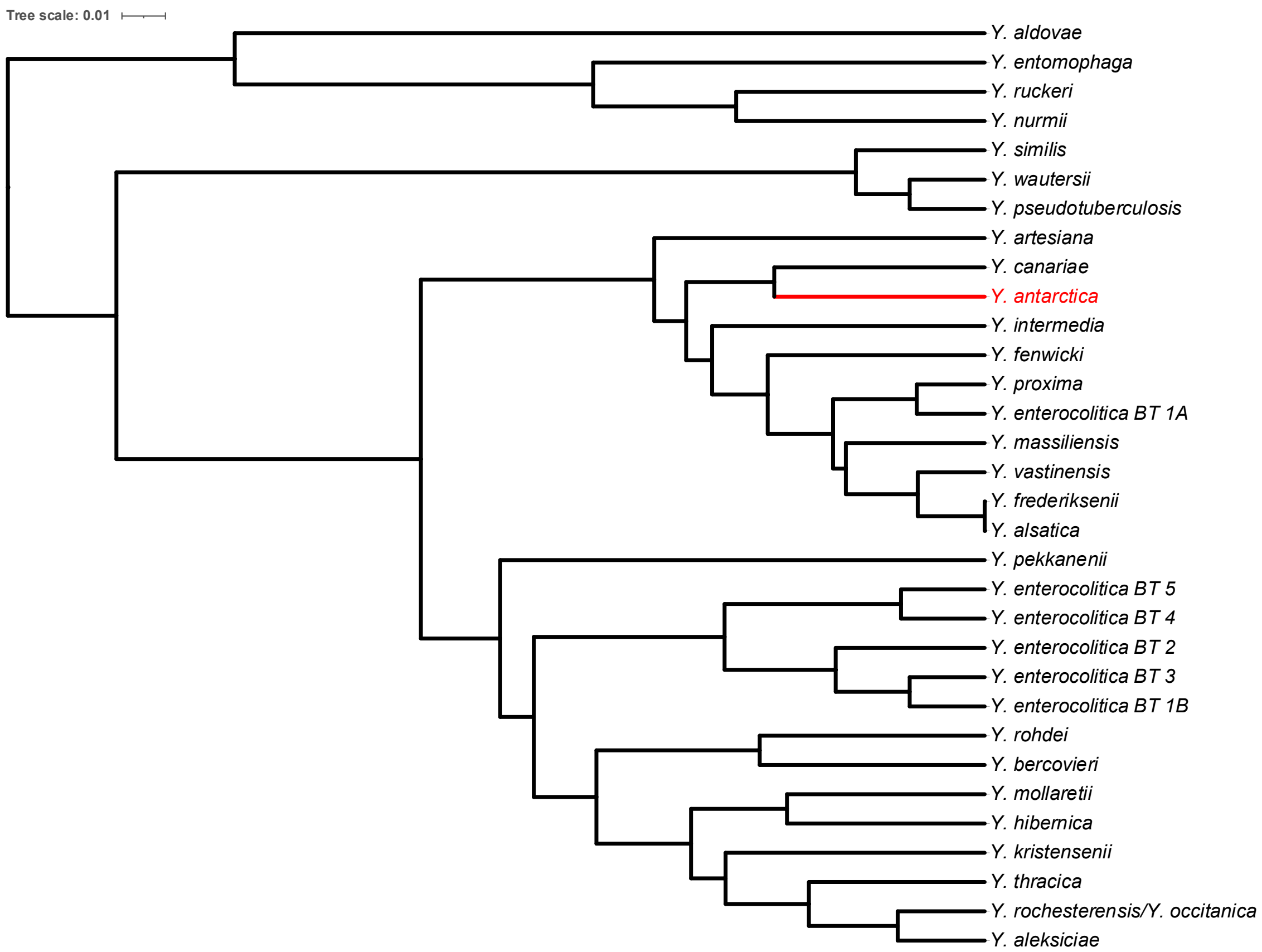

Legend: Unweighted Pair Group Method with Arithmetic Mean (UPGMA) dendrogram based on biochemical characterization of 27 *Yersinia* species species (Table 2). The dendrogram was constructed using the UPGMA based on a Manhattan distance matrix calculated from 36 biochemical characters. Each character was scored as positive (1), variable (0.5), or negative (0). Distances were normalized by the total number of characters. Clustering analysis was performed using R software (v4.4.2) with the ape package (v5.8-1). The Newick tree was visualized using iTOL v7 [31].

Figure S2:

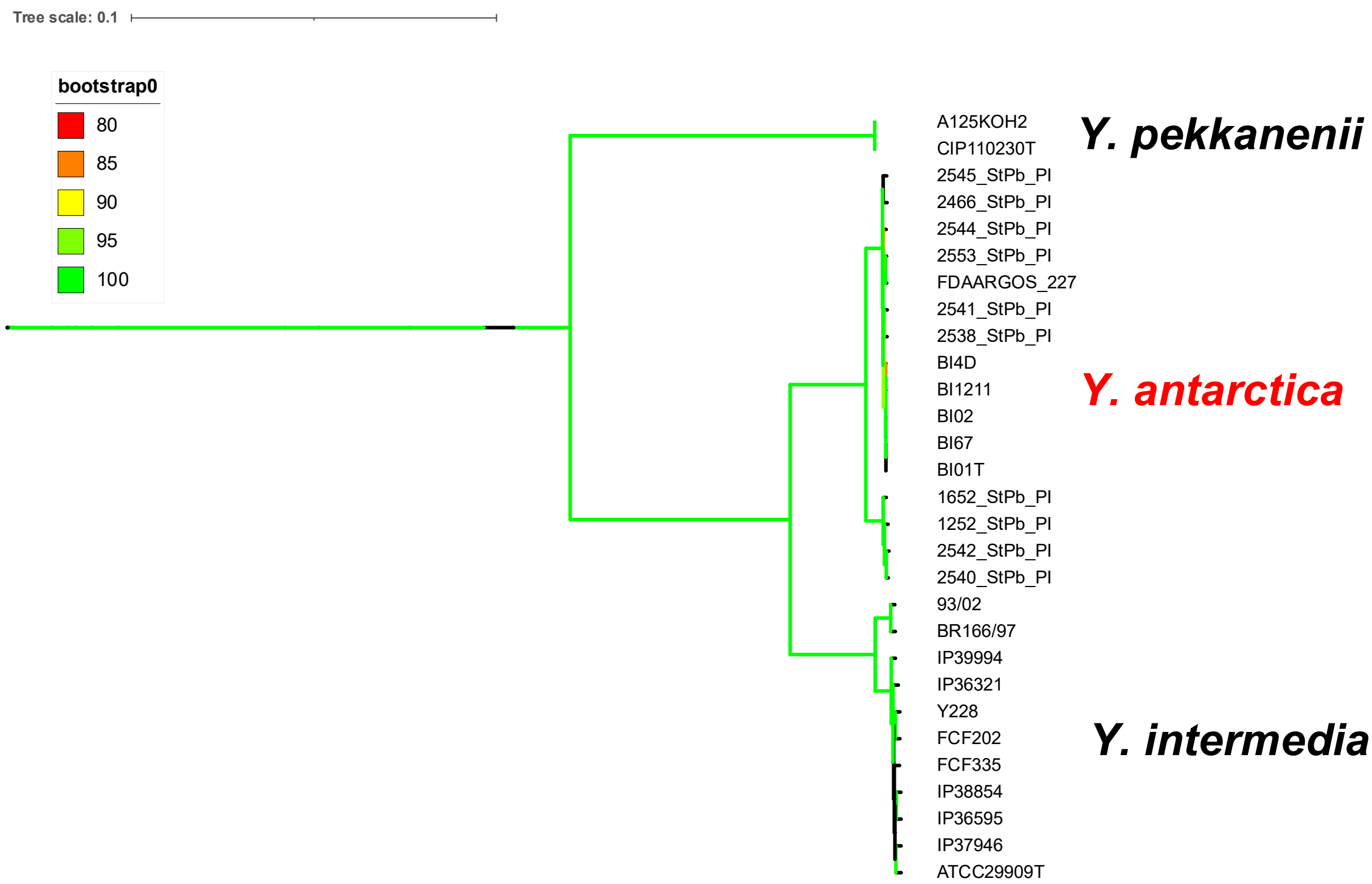

Legend: Maximum-likelihood phylogenetic tree of the evolutive branch (29 strains) encompassing *Y. pekkanenii*/*Y. antarctica*/*Y. intermedia* based on 500 concatenated genes multiple sequence alignments. Tree visualization was created with iTOL v7 [31]. Boostrap support values are depicted using a color gradient. The clade corresponding to "*Y. antarctica*" is highlighted in red.

Figure S3:

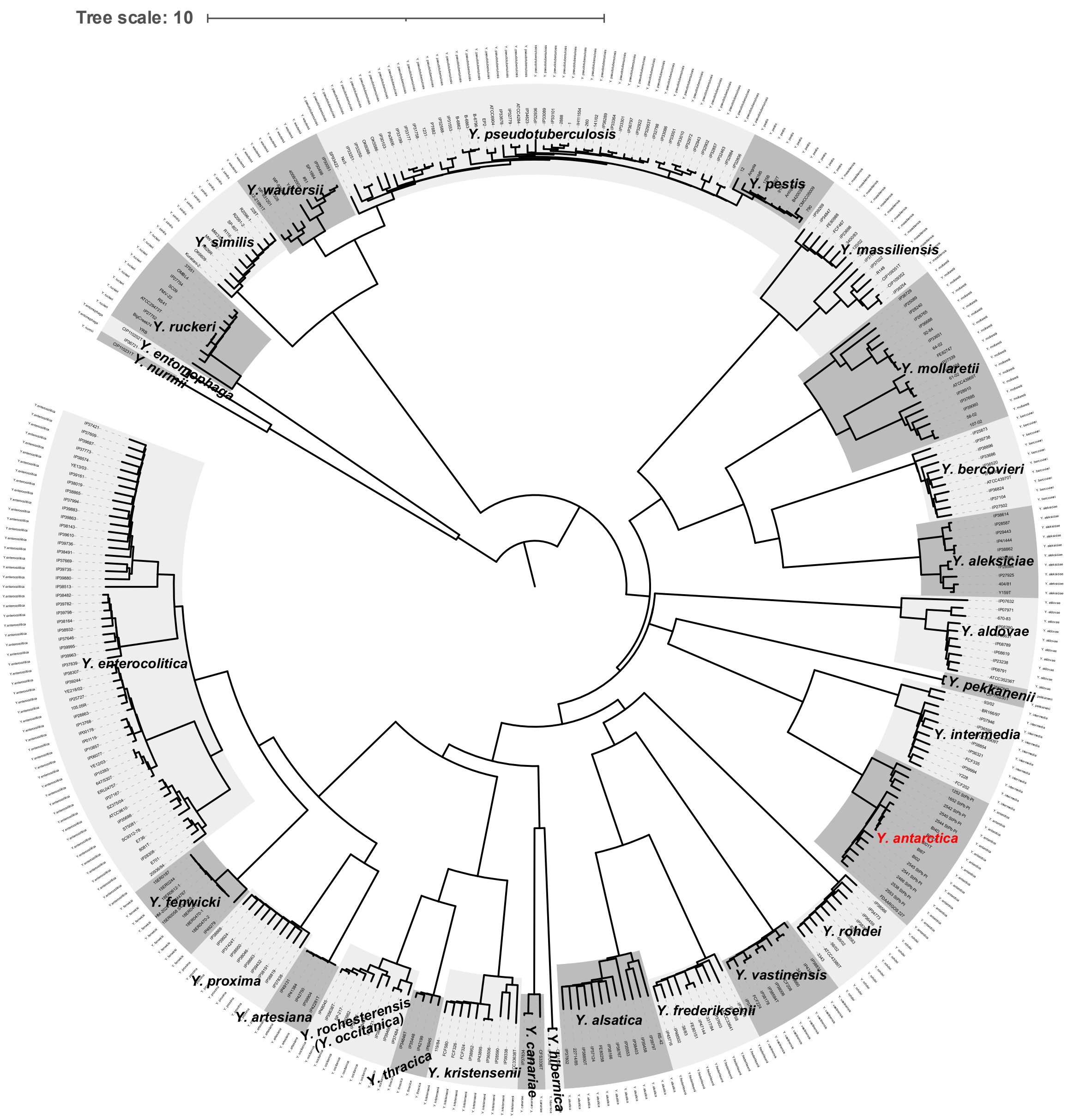

Legend: Neighbour-joining tree generated based on the ANI matrix (Table S2) with 333 *Yersinia* strains, using ape package (v5.8-1) installed with R (v4.4.2). Tree visualization was created with iTOL v7 [31]. The clade corresponding to "*Y. antarctica*" is highlighted in red.
